# Supplementary material for: Signal, noise, and sampling: how pool size and replication shape metabolomic inference
Source: G3 (Bethesda). 2026 Sep 2;16(11):jkag249. doi: 10.1093/g3journal/jkag249 (PMC13732477; doi:10.1093/g3journal/jkag249)
Supplement: Supplementary file 1 [file jkag249_supplementary_data.zip › Figure_S1_legend_G3-2026-407137.docx]

**Figure S1. Per-metabolite pool-size sensitivity.** Each point is one metabolite; y-axis shows the absolute t-value for the log(pool size) effect after accounting for diet. Point color indicates direction of the pool size effect. Dashed line = |t| = 2. (Supplemental Table 6D)
